# Identification at Biovar Level of *Brucella* Isolates Causing Abortion in Small Ruminants of Iran

**DOI:** 10.1155/2012/357235

**Published:** 2012-11-27

**Authors:** Ali Mohammad Behroozikhah, Ramin Bagheri Nejad, Karim Amiri, Ali Reza Bahonar

**Affiliations:** ^1^Brucellosis Department, Razi Vaccine and Serum Research Institute, Karaj 3197619751, Iran; ^2^Office of Brucellosis Control and Eradication, Iranian Veterinary Organization, Tehran 1595814111, Iran; ^3^Food Hygiene and Quality Control Department, Faculty of Veterinary Medicine, University of Tehran, Tehran 1419963111, Iran

## Abstract

To determine the most prevalent biovar responsible for brucellosis in sheep and goat populations of Iran, a cross-sectional study was carried out over 2 years in six provinces selected based on geography and disease prevalence. Specimens obtained from referred aborted sheep and goat fetuses were cultured on *Brucella* selective media for microbiological isolation. Brucellae were isolated from 265 fetuses and examined for biovar identification using standard microbiological methods. Results showed that 246 isolates (92.8%) were *B. melitensis* biovar 1, 18 isolates (6.8%) were *B. melitensis* biovar 2, and, interestingly, one isolate (0.4%) obtained from Mazandaran province was *B. abortus* biovar 3. In this study, *B. melitensis* biovar 3 was isolated in none of the selected provinces, and all isolates from 3 provinces (i.e., Chehar-mahal Bakhtiari, Markazi, and Ilam) were identified only as *B. melitensis* biovar 1. In conclusion, we found that *B. melitensis* biovar 1 remains the most prevalent cause of small ruminant brucellosis in various provinces of Iran.

## 1. Introduction

Brucellosis is a worldwide bacterial zoonosis posing hazards to the public health and causing economic losses for livestock production industry [1–3]. Animal brucellosis is mainly characterized by reproductive involvement resulting in abortion and infertility [2], whereas human brucellosis is a febrile illness known as undulant fever which can lead to chronic debilitating complications [3, 4]. The disease results from facultative intracellular bacteria belonging to the genus *Brucella* which are Gram-negative, nonspore-forming and noncapsulated coccobacilli [3, 5, 6]. Currently, the genus consists of 10 species classified based on their host preferences and phenotypic differences [2, 7]. Three species are also divided into biovars which are *B. abortus*, *B. melitensis,* and *B. suis* with 7, 3, and 5 biovars, respectively [3].

In sheep and goats, brucellosis is primarily due to *B. melitensis* which is the most pathogenic species for human beings, responsible for the main proportion of human cases in endemic regions including Mediterranean and Middle East countries [1, 3, 8–10]. Humans catch the disease via direct contact with infected animals, their vaginal discharges, and aborted fetuses; dealing with pure cultures; and consumption of unpasteurized contaminated milk and milk products [3, 6, 11]. The latter is the major way through which *B. melitensis* is transmitted to people in endemic areas [1, 5]. 

Identification of *B. melitensis* biovars involved in small ruminant brucellosis is a critical component of epidemiological surveys required for designing proper preventive and control strategies [8]. In Iran, due to the existence of other related factors including illegal livestock import from neighboring countries where *B. melitensis* is also prevalent and uncontrolled animal movements, it was considered necessary to study changes in the epidemiologic distribution of different biovars in order to determine introduction of new biovars and evaluate the success of ongoing control measures. Therefore, the present study was performed to renew our knowledge about the current prevalence status of *B. melitensis* biovars causing abortion in sheep and goats in Iran.

## 2. Materials and Methods

### 2.1. Study Design and Sampling

The present cross-sectional study was carried out from 2007 to 2009. According to Iranian Veterinary Organization classification, all country provinces were divided into 3 categories with low (<2%), intermediate (2-3%), and high (>3%) brucellosis prevalence. This classification is based on a seroepidemiological survey carried out in 2003 using conventional rose bengal, serum agglutination, and 2-mercaptoethanol tests. Two provinces were then selected from each category based on geography and availability of a central laboratory in the Provincial Veterinary General Office with skilled personnel for bacterial isolation. The selected provinces were Kerman and Markazi; Ilam and Khorasan Razavi; Chehar-mahal Bakhtiari and Mazandaran from low, intermediate, and high prevalence categories, respectively. All small ruminant aborted fetuses referred to the central laboratory of The Provincial Veterinary General Office during the study period were cultured for brucella isolation. Brucella isolates were sent to Brucellosis Department of Razi Vaccine and Serum Research Institute to be identified at species and biovar levels.

### 2.2. Isolation of Brucellae

Specimens obtained from liver, lungs, spleen, and fetal stomach content of referred aborted fetuses were cultured on brucella agar (BD, USA) containing 5% (v/v) inactivated horse serum, 2% (w/v) dextrose, and brucella selective supplement (Oxoid, UK) according to the manufacturer's instruction. Tissue specimens were first decontaminated using ethanol and flame and then homogenized in sterile normal saline by means of a grinder. For each specimen, two sets of 3 agar plates were inoculated and incubated at 37°C with one set in the air having 10% CO_2_. Inoculated plates were monitored every 2 days to detect any colonial growth, and in the case of no colony observation, they were kept at most for 35 days. Suspected colonies based on their morphological characteristics were subcultured on brucella agar slopes for further evaluation and biovar identification.

### 2.3. Biovar Identification

Brucella isolates, confirmed by microscopic characterization using Gram stain and results of catalase, oxidase, and urease tests, were examined for biotyping according to the standard methods described by Alton et al. [12]. Briefly, biovars were identified based on agglutination with A and M monospecific antisera, CO_2_ requirement for growth, H_2_S production, lysis by Tbilisi (Tb) and Berkeley (Bk_2_) phages, and growth on media containing 20 *μ*g/mL basic fuchsin and thionin dyes [12–15].

## 3. Results

In the present survey, a total number of 851 aborted sheep and goat fetuses were cultured microbiologically and 265 brucellae were isolated. Biotyping of these 265 isolates showed that 246 (92.8%) were *B. melitensis* biovar 1. Eighteen isolates (6.8%) were identified as *B. melitensis* biovar 2; and interestingly, one isolate (0.4%), which was obtained from Mazandaran province, was determined as *B. abortus* biovar 3.  Table 1 illustrates numbers of brucella isolates attained in different provinces and results of their biotyping.

*B. melitensis* biovar 3 was isolated in none of the six provinces during the study period, and *B. melitensis* biovar 2 was only isolated in two provinces which are Khorasan Razavi and Kerman belonging to intermediate and low prevalence categories, respectively.

## 4. Discussion

Small ruminant brucellosis is still a major animal and public health burden in many parts of the world particularly in the Middle East and Mediterranean Region [1, 6, 8, 10]. Sheep and goats are primarily infected by *B. melitensis* as its preferential hosts [8, 18]. Regarding the public health, *B. melitensis* is the most important zoonotic pathogen amongst the *Brucella* spp. [1, 3, 5], accounting for the vast majority of human cases all around the world [8]. In endemic areas, it is transmitted to people mostly through consumption of unpasteurized milk and milk products from sheep and goats [3, 5, 19].

*B. melitensis* is divided into three biovars which are differentiated using conventional laboratory methods such as agglutination with A and M monospecific antisera and lysis by brucella phages [12, 13]. Determination of biovars involved in animal brucellosis is an important step for epidemiologic characterization of the disease in any country and a preliminary requirement for designing control and eradication programs [8]. Additionally, owing to the ability of brucellae to adapt to new environments and its re-emergence [3, 5, 6], revealing changes in epidemiologic features of *Brucella* species/biovars can help to unravel complexity of interactions between the organism, animals, and humans [5].

In Iran, the first *B. melitensis* isolation from an aborted sheep fetus was reported in 1950 [16, 17]. Thereafter it has been widely isolated in different parts of the country mainly from sheep and goats but also occasionally from cattle, camel, and sheepdogs [17, 20]. All biovars of *B. melitensis* exist in Iran among which biovar 1 is known as the most widespread.  Table 2 summarizes biotyping results of Iranian *B. melitensis* isolates during two periods of time from 1971 to 2000.

Immunization of host species with *B. melitensis* strain Rev.1 has been the main strategy for the control of small ruminant brucellosis in Iran since 1960s. During 1983–2003, a test-and-slaughter campaign was also conducted in adult sheep and goats using rose bengal, serum agglutination, and 2-mercaptoethanol tests, while vaccination was limited to young animals. From 2003, control program has been based on mass vaccination using full doses (containing 1–3 ×10^9^ bacteria per dose) of Rev.1 vaccine in lambs and kids at 4–7 months of age, and its reduced doses (containing 0.5–2 ×10^6^ bacteria per dose) in adult female animals. In addition to the mass vaccination, other measures including public education, promotion of sanitary husbandry practices, and microbiological evaluation of abortion outbreaks have been implemented. As a result, the number of new human cases reported annually dropped from 39 in 2005-2006 to 15.9 in 2010-2011 per one hundred thousand people in the country (unpublished data).

Our survey showed that *B. melitensis* biovar 1 is the most frequent cause of clinical brucellosis in small ruminant populations of the provinces included. This finding is in agreement with the results of the previous studies in the country [16, 17]. Isolation of *B. abortus* biovar 3, which is an enzootic etiologic agent of bovine brucellosis [17, 21, 22], from an aborted sheep fetus in our study suggests the possibility of cross-species transmission of the pathogen from cattle. *B. abortus* has been sporadically identified and reported as a causative agent for sheep brucellosis in Iran [17, 21]. This indicates that in endemic areas, where both *Brucella *spp. are present, and cattle and small ruminants are raised in close contact, transmission to nonpreferred hosts may occur [17, 21, 23]. This should be taken into account while implementing control measures.

Whereas the estimation of disease prevalence was not the aim of this study, the results showed that in spite of implementing vaccination and other control measures for years, small ruminant brucellosis in clinical form still persists in various parts of the country. Nevertheless, there is a need for country-wide investigations covering the whole target population including traditionally and nomadically reared flocks [6] to ascertain geographical distribution of different biovars region by region. It will also help trace potential outbreaks, especially in provinces neighboring western and eastern borders. For this purpose, using geographic information system (GIS) will be helpful in analyzing interactions between animal and human brucellosis [24].

## 5. Conclusion

The present study revealed that *B. melitensis* biovar 1 remains the most prevalent cause of clinical form of small ruminant brucellosis in various provinces of Iran. However, more studies are required to determine current status of the disease throughout the country and the evaluation of its impact on human health. Further control and prevention measures can be implemented to curtail the human and animal disease incidence to a greater extent.

## Figures and Tables

**Table 1:** Number of isolates in different provinces.

Province	Prevalence category	Number of isolates	Number of different species/biovars		
			*B. melitensis *	*B. melitensis *	*B. abortus *
			biovar 1	biovar 2	biovar 3
Mazandaran	High	55	54	—	1
Chehar-mahal Bakhtiari	High	58	58	—	—
Khorasan Razavi	Intermediate	68	55	13	—
Ilam	Intermediate	10	10	—	—
Markazi	Low	39	39	—	—
Kerman	Low	35	30	5	—

Total (%)		265 (100)	246 (92.8)	18 (6.8)	1 (0.4)

**Table 2:** Frequency of different *B. melitensis* biovars isolated in Iran.

Years	*B. melitensis *	*B. melitensis *	*B. melitensis *	Total	References
	biovar 1	biovar 2	biovar 3		
1971–1980	851	242	4	1107	[16]
1981–2000	2102	205	106	2413	[17]

Total	2953	447	110	3510	
